# The Accoucheur; a Treatise on Protracted Natural Labours, Suspended Animation in New-Born Infants, Uterine Hemorrhage, &c.

**Published:** 1839-10

**Authors:** 


### ART. XI.

*The Accoucheur; a Treatise on Protracted Natural Labours, Suspended Animation in new-born Infants, and Uterine Hemorrhage after birth of the Child; with illustrative Cases.* By JOHN CRAIG, Surgeon, Paisley.—*Glasgow*, 1839. 12mo, pp. 252.

THE contents of this work are enumerated in the title-page. In his preface, the author offers advice to, and passes some observations upon, his medical brethren which cannot be called flattering: "However much," he says, "our professional friends may be opposed to innovation in the practice of their art, without the sanction of some great name, we urgently ask them simply, in the first place, to read and study our small volume; then we doubt not they would enjoy, perhaps for the first time in their lives, what ought always to stand with medical men in submitting their observations and reflections to practice, the conscientious approval of their own minds. But from the few testimonies we have quoted as well as from our own personal knowledge, we have misgivings regarding our medical brethren." (pp. viii, ix.) Such then is the lamentable state of midwifery that as yet we have seldom or never felt conscientiously satisfied with the results of our practice: how comfortable must the opposite condition be which is enjoyed in so eminent degree by the author! The work is indeed characterized throughout by unbounded assurance and self-esteem, and it is with unfeigned regret that we are compelled to notice in it also something of that invidious and disingenuous spirit which has marked some other obstretrical writings that have recently proceeded from the northern press. At the beginning of the very page from which we have made the above quotation, an insinuation is thrown out that Dr. Churchill of Dublin is one of "those who scarcely know what improvement means." Against such a charge we must enter our protest. Although in our review of Dr. Churchill's work on the Diseases of Females (*B. and F. M. R.* July, 1838,) we ventured to point out a few omissions or defects, yet in common fairness to its learned author we cannot let such an assertion pass without exposing its injustice. In the work in question Dr. Churchill has proved himself to be a man of very extensive reading and also ardently devoted to the practice of his profession.

Mr. Craig begins his first chapter on "protracted natural labours" with the following definition: "Under the designation of protracted natural labour are comprehended those forms in which the pelvis is of such dimension and capacity as to allow a child of the usual size to pass, and when the presentation is of such a kind as to permit the passage by the ordinary efforts of the expulsive powers, but in which from some other obstructing cause the period of delivery is delayed." (p. 1.) Where the protraction of labour neither depends upon the pelvis nor the child, it must arise from one of two other sets of causes, viz., a faulty condition of the soft passages or of the expelling powers. Mr. Craig's observations refer only to derangements of the expelling powers, but he must be aware that the above definition, as he has stated it, evidently refers to the other species of dystocia also. We object to the term "protracted *natural* labour." The cases quoted by him are *very* far from being normal, or consonant with a natural course of parturition; they are cases of extreme suffering, difficulty, and considerable danger; and we are surprised at the present day to see that, in conjunction with several of his countrymen, he still continues to use the old and faulty classifications of labours.

Mr. Craig sets out with the conviction that midwifery practitioners have hitherto been in the habit of using the perforator unnecessarily, and believes that if they would only read his "Novum Lumen Obstetricantribus," they will see the error of their ways. We might excuse this display of self-importance, although it pervades the work rather extensively; but we cannot pass over the sneering remarks which are made upon authors who have published the results of their own large experience; the more so as the real state of facts is sometimes considerably warped and perverted, we trust unintentionally.

"In the Maternité at Paris, the perforator was used only sixteen times in upwards of 20,000 cases, and the celebrated Dr. Dewees of Philadelphia in more than 3,000 deliveries did not use it once. . . . . Dr. Merriman, Dr. Collins, and many others found it necessary to employ the perforator more frequently than the above authorities. Dr. Merriman in 2947 cases used the perforator nine times, and Dr. Collins in 16,654 cases employed it 118 times. So frequent an application of so fatal an instrument by so distinguished an accoucheur as Dr. Collins startles us not a little, particularly when we are told that it was employed more frequently than the forceps. Although Dr. Collins has put his mode of practice on record, and although it is favorably noticed by the medical periodicals, yet we trust that neither he nor any one else will long continue the same course." (p. 6.)

Now this statement implies a severe censure upon the professional conduct of two gentlemen who have attained that high distinction which is so eminently due to them. If the author had read our review of Dr. Collins's work, (*Br. and For. M. R.*, Vol. II., p. 77,) he would have seen (if he had never known it before) to what circumstances is the practice at the Maternité indebted for this unusually rare use of the perforator. If a statement of this sort is to be made, the whole of it should be honestly stated and not a portion of it kept back. There is no doubt but that in many cases where the forceps has been used in Paris, the perforator ought to have been employed. The forceps used there is of great length and power, and we have no hesitation in saying that the child may by it be dragged through a narrow pelvis with a degree of force which nothing can justify. Mr. Craig has taken the statement of Dr. Merriman's practice from the work on difficult parturition by this distinguished author, and as the statistical table of the Maternité precedes Dr. M.'s report of his own practice, Mr. Craig must necessarily have read it, if he did not actually quote it from that work: candour therefore should have suggested the propriety of adding the observations which Dr. Merriman has appended at the end of this table. "No mention," says Dr. Merriman, "is made of the number of deaths among the children born without artificial assistance, but among the 334 where artificial aid was required, 91 were dead born, of which 68 appear to have lost their lives during labour and 23 were dead before the labour began: of the deaths of the mothers we learn nothing from Madame Boivin."* It would have been much more fair and candid to state the nature of the nine cases in which Dr. Merriman "found it necessary to employ the perforator" than to insinuate the charge of mal-practice by trusting that neither Dr. Collins "nor any one else will long continue the same course." Every impartial mind on referring to Dr. Merriman's table of his own practice will at the first glance justify him in his use of the perforator. Of the nine cases (or one in 328) it was used seven times from distortion of the pelvis, in four of which cases premature labour was afterwards induced, and two of the children saved; in the remaining two cases it was used "in very lingering labour, *when the want of pulsation in the presenting funis had fully proved the death of the children.*"† And yet with all this staring him in the face, the author ventures to make the following observation: "To what degree the distortions extended is not so much as hinted at, but from what we have stated above and from our own experience we can scarcely admit that they could be all of the description to prevent a

* Synopsis of Difficult Parturition. Table ii, p. 327. 1838. † Op. cit. Table vii, p. 336.

child of the usual size to pass with strong efficient pains. Be this as it may, two of the children's heads were perforated on account merely of the labours being very lingering!" (p. 7.) We would in the first place ask what right has the author or anybody else to doubt the word of such a man as Dr. Merriman? Or on what grounds does he suppose that the perforator was *not* fully justified in these cases? In the two others, where the funis presented and the child was already dead, the author is guilty of misrepresentation (we still hope unintentionally) in saying that the perforator was used "on account merely of the labours being very lingering." The attack on Dr. Collins is still more personal, and what is worse, the numbers are not even stated correctly. Of the 16,654 births recorded by that gentleman, *seventy-nine* (not 118) children were delivered by the perforator, and yet with this diminished proportion, viz., one in 210 cases, it is unfair to state even these numbers without making the reader in some degree aware of the circumstances under which they occurred. "In this report," says Dr. Collins, "of the number of children delivered by the crotchet, it is necessary to bear in mind that the *proportion* of such deliveries is greatly increased in consequence of the *same patient* returning to the hospital *two*, *three*, or even *more* times, in whom from deformity or other circumstances such mode of delivery was rendered unavoidable. . . . . Other circumstances occurred where delivery was effected by lessening the head, viz., in rupture of the uterus, prolapsus of the funis where the child was dead, and convulsions; these cases are recorded under the several heads stated. In my opinion the operation under the latter circumstances is altogether different, as in such the head is lessened in order to effect delivery with the least possible risk to the mother, without trusting longer to nature's efforts owing to *immediate* and *extreme* danger to the patient, or to prevent her suffering *unnecessarily* when the child is dead."* Mr. Craig should also have mentioned a fact connected with Dr. Dewees's practice which in great measure accounts for his not having used the perforator. "The occurrence of deformity of the pelvis in this country is so very rare as not to have been even encountered by some practitioners of pretty extensive experience; as far as regards our own, we must declare that we have not met with extreme deformity in American women three times in our lives; and when it has occurred to such extent as to render labour impracticable by the natural powers, it has uniformly been with European women."† We must apologise for devoting so much time and space to points which some of our readers might suppose of comparatively slight importance; but in our capacity of reviewers we cannot pass over observations like the above without deprecating the spirit in which they are made, whatever may have been the author's precedent for doing so.

The author enumerates four causes of protracted labour: the first "is inflammation, the second is congestion, the third an excess of sensibility, and the fourth is spasmodic." (p. 20.) We do not object to them, for there is nothing novel in the fact that such conditions frequently interfere with the regular action of the expelling powers. Why the author should have been so many years, as he informs us, in "ultimately" coming to

* Practical Treatise on Midwifery, by Robert Collins, M.D. pp. 487, 8.
† Compendious System of Midwifery, § 37.

this conclusion, we know not; for it was one of the earliest practical facts that was impressed on our mind as a student. In his sixth section, on the "Symptoms that indicate the causes of protraction," those of the inflammatory state are fairly enough, although briefly, given; they are well known; those, however, of congestion, at least as given by the author, are, perhaps, somewhat novel.

"In patients labouring under this form of obstructing cause, the skin is below the natural standard of heat; pulse nearly natural, both in strength and in the number of beats; little thirst; some appetite, at least at the commencement of labour. Throughout this form of labour the patient manifests considerable reluctance to motion; and as the labour advances, she becomes a torpid, unwieldy mass, scarcely able to assist herself to turn in bed or come out of it. After the labour has continued a few hours, the lower part of the belly becomes hard and tender to the touch; and during the pains the woman utters a dull but plaintive cry. Little urine forms, and the patient has to be urged to pass it. Vagina and os uteri generally cool to the touch and free from pain. Bowels generally constipated. Pains not frequent, at least at the beginning of labour, but they are very distressing to the patient, and advance the child but very little. It deserves notice that in labours of this description, and sometimes in the former, there are, during the intervals of regular pains, severe grinding pains in the lower part of the belly." (p. 23.)

The first part of this description conveys no information, and the latter part merely shows the presence of what are commonly called spurious pains. His description of the other causes of protraction is equally vague and unsatisfactory, and betrays a very confused view of the subject, although the whole is laid down with a degree of self-importance which is amusing.

The author's treatment is simple: it consists in giving the patient a drachm of laudanum at a dose, and repeating it twice more if necessary. If there is inflammatory action, he bleeds; if the bowels are constipated, he gives an enema of common salt and butter in warm water; and if all this fails, he gives the ergot of rye. The effect of this treatment in the majority of cases is to excite very severe but inefficacious pains, putting the patient to intense suffering, and, in some cases, driving her to a state of excitement verging on delirium. We have perused the author's cases carefully and impartially, and can come to no other conclusion than that the uterine action had been most improperly interfered with by the unjustifiable use of over doses of opium; and that in several instances the bleeding and enema were indicated solely to calm the excitement which had been produced by the opium. We can speak from pretty large experience on these matters, and can safely affirm that we seldom or never meet with cases of such violent, irregular, and ineffective uterine action where there had not been gross mismanagement and interference; wherever this state has been induced it has been capable of being restrained by the proper use of the lancet, enemata, and the warm-bath. Opiates here we have found decidedly useful, but not in the form or dose recommended by Mr. Craig. Under no circumstances do we venture to give opium during labour, without combining it with a diaphoretic, either in the form of Dover's powder, or in a draught with antimonial wine. When a gentle equable perspiration is induced by these remedies, it brings great relief to the patient and effects a complete change in the whole character of the labour. Thus combined, and in moderate doses, we find opium an admirable remedy: it allays irritation and induces sleep, from which the patient awakes astonishingly refreshed. It is now that, with the moisture upon the skin, we feel the pulse has become slow and soft; the vaginal secretion copious, the os uteri soft, cushiony, and dilatable, not thin and dilatable, as the author has expressed himself, for such a state can scarcely exist. The author himself admits, and his cases prove it, that the laudanum does produce the injurious effects which we have attributed to such over doses.

"For in general those cases which really require the employment of laudanum are too obstinate to be effectually relieved by any single means; and we find that, after the first dose of the opiate has been administered, the pains continue unchanged in regard to their efficiency, though in severity they are often increased. Under such circumstances, about an hour after the first dose of laudanum was administered, a second and similar dose is given to the patient; and, should this second quantity fail, in about half an hour after it has been swallowed, to produce a sensible improvement on the labour, we may rest assured that the inflammatory and other symptoms will require bloodletting, in order to remove their obstructing influence over the expulsive efforts of the mother, and to bring the labour to a speedy termination." (p. 39.)

Mr. Craig's remarks on the action of the enema are very sensible and just, but they contain nothing which is not familiar to every practitioner in midwifery. No one has pointed out the use of enemata in labour more fully than Mauriceau.

Mr. Craig states that, "in the treatment of protracted natural labours, we are uncompromising advocates for shortening their duration," (p. 37;) and he limits this duration, "in almost every instance," to "within or in about twelve hours." (p. 50.) "We have always," says he, "practised the manual assistance mentioned by Dr. Hamilton, and have never hesitated to introduce the point of the finger within the os uteri, moving it gently round within its orifice." (p. 57.) All this we object to strongly; it shows that the author must have a very indistinct knowledge of the real process and mechanism of natural labour. It is to those who entertain such views that the celebrated Naegelé refers when he says, "instead of studying the laws of nature by long and laborious observation, they presumed to dictate to her."* We are the more astonished at these views, as we know how admirably the subject has been handled by Boer, Wigand, &c. in Germany; by Denman, Merriman, &c. in England; and so lately and successfully by Dr. Collins, E. Murphy, Churchill, &c. in Dublin.

The author's views respecting the use of the forceps are none of the clearest. He does not profess to know anything about the stethoscope, as applied to midwifery, and therefore considers it useless.

In stating the duration of some of his labour cases, the author does not appear to be very accurate as to the precise time; thus, in case xii., the patient, considering herself in labour, requested him to call early in the forenoon, when she stated that she had been suffering pains for some hours previously; the os uteri was dilated the size of a shilling, and the presentation so high up as to be reached with difficulty. Having made her swallow two drachms of laudanum, and the pains being extremely violent, although ineffective, bleeding became necessary; the third drachm was also given, and, after a dreadful night of pain and vomiting, the

* Mechanism of Parturition, Transl. p. 3.

child was born at five in the morning, as the author coolly assures us, "ten hours after the labour had actually commenced!" (p. 121.) Some of the cases seem to us striking examples of the mischievous effects of Mr. Craig's peculiar treatment. Case viii. was of a patient in delicate health and in her seventh pregnancy; on being called to her the author found that she was suffering under spurious pains, and gave her a drachm of laudanum. She "vomited repeatedly during the night," but was easy the next morning. On the following morning "the pains appeared of a genuine description"—"the os uteri was but little dilated, yet the pains had the effect of rendering its edges tense and its orifice more capacious." "During the subsequent two hours after our arrival at the patient's house, the pains continued frequent and severe, yet at the end of this period no perceptible change had taken place on the os uteri. As she had now been about three hours in labour, sixty drops were administered with a view to modify the unavailing uterine actions; and, as no alteration whatever took place at the expiration of an hour after the laudanum was given, a second and similar dose was exhibited. These two full doses of laudanum had no effect in abating either the frequency or violence of the pains, and, although we waited for an hour after the second dose was given, in expectation of a favorable change, yet the pains continued as inefficient as at the commencement of labour. The patient by this time was suffering under most agonizing pains, producing slight delirium; she could scarcely move in bed; belly hard and painful, considerable heat of skin, pulse accelerated." (p. 109.) Bleeding had of course become necessary, by which means the pains became efficient, and the child was born just in time to save her from (what the author calls) a *third* dose of laudanum. If in this case the bowels had been thoroughly evacuated, instead of a drachm of laudanum being given, at the beginning of the labour, and if this unjustifiable interference had not been repeated, we have little doubt but that the course of labour would have been very different; a mild diaphoretic after the action of the laxative would have assisted still further in rendering the pains effective, and in preventing any disposition to inflammatory action.

Case xiv. is a dreadful detail of agony and, we think, malpractice; nor can it be palliated by the circumstance of the patient being an opium-eater; it indeed merits the epithet *instructive*, which, in one respect, applies to the whole book; it should be a warning to all practitioners against being carried away by false theories and blind prejudices. The os uteri had dilated to the size of half-a-crown, between four and five o'clock in the afternoon. "By eleven o'clock the pains had become very severe, but did not advance the child. This being the usual time that she was in the habit of taking a second opium pill, one of two grains was administered, but it had no effect in alleviating her sufferings or improving the labour. She continued in this state until half past twelve o'clock, when her sufferings became so intolerable that she exclaimed it was impossible she could longer support such agony. The purgative clyster was now administered, which operated well; and after its operation, sixty drops of laudanum were given to the patient. In about half an hour after these means were employed, the pains became less severe, and more effective; and by two o'clock, A.M., the child's head had advanced so far as nearly to dilate the external parts of the mother. From this period until three o'clock no change took place, except the pains being more aggravated. The patient now lay on her bed like an inflexible mass of matter, being incapable of motion without assistance; her belly, particularly that portion of it situated over the fundus of the uterus, was so hard and tender that she could scarcely bear it to be touched." (p. 126.) Bleeding, of course, became necessary; and as a last goad to exhausted nature, the ergot of rye was given, and the child at length expelled; the author does not think fit to inform us *when* this event really took place, but merely states that the patient "was little more than twelve hours in actual labour." Almost every case is described as being attended with the most violent and agonizing uterine action, which, if not caused, must, we think, have been at least severely aggravated, by these doses of laudanum.

In his fifteenth case we have the following remark: "The expelling efforts, however, soon ceased, or at least were completely counterbalanced by the resisting powers. The practice now to be pursued was very evident: that of improving the expulsive efforts by the ergot of rye." (p. 131.) We, of course, do not object to this reasoning, but it comes rather oddly to the reader after the following passage, a few pages back. "The nature of the case, the amount of remedial means used, and the stage of the labour, showing that the expulsive powers predominated over those of resistance, clearly evinced that the only safe course to pursue was to increase the expulsive powers of the uterus by means of the ergot of rye." (p. 118.)

The third chapter is "on suspended animation in new-born infants," and is marked with the author's characteristic faults. He appears to be ignorant of the two forms under which *asphyxia neonatorum* is usually described—the one a state of congestion, the other of atony and exhaustion. He remarks that, "from the relaxed and shrunk appearance which many, if not all stillborn children exhibit, one is apt to imagine that the child has continued to be emptied of its blood by the umbilical arteries, after it had ceased to receive any by the umbilical vein." (p. 137.) On this observation we remark that, in the first place, a large proportion of stillborn children present a very opposite appearance to that just mentioned, viz. one of general congestion; and, as to the child being "emptied of its blood by the umbilical arteries," we should like to know where the blood goes to? The author neither uses frictions with spirit nor the warm bath, which are looked upon by most practitioners as such valuable remedies in these cases; he places his chief confidence in inflation of the lungs, and continues this operation even for some time after the child has begun to breathe. In speaking of this operation he makes an excellent remark, which, although not new, is not the less valuable, and is not sufficiently attended to.

"It deserves to be particularly noticed, too, that when the child begins to show symptoms of returning animation, its tongue will sometimes be so forcibly retracted, as to fill up the passage of the throat so completely as to render the inflation of the lungs very difficult; indeed, its accomplishment, in some cases, is quite impossible, until the tongue is drawn forward or pressed down by the finger, so as to allow the air to pass. The same thing occasionally takes place even after the child has respired naturally. We have felt considerable embarrassment in the management of such cases, for, after respiration had been fully restored, all at once the breathing ceased; and, on endeavouring to inflate the lungs, not a particle of air passed down the trachea.

On examining the throat, by means of the finger, the tongue was found drawn backward and rigidly fixed, so as completely to shut the rima glottidis. To remedy this evil, we introduced our fore-finger back to the root of the child's tongue, and pressed it gently forwards and downwards, when the opening became so far relieved as to allow the air again to pass, and the child soon began to respire." (p. 144.)

Case v. illustrates, we think, the impropriety of not using the warm-bath and external stimulants. The child was stillborn; there was slight pulsation in the chord; the face and body were livid and shrunk, but, on inflating the lungs, they became of a florid colour, and the heart began to pulsate strongly. The inflation was continued for at least nine hours, during which time, the author remarks, "we never observed the child to make the least effort to breathe, neither was any external motion observed in any part of the body; yet all the time the heart continued to beat, and, for the most part, the lips and face were of a florid, healthy-like colour." (p. 147.) At the expiration of this period the colour again became dark, and the heart ceased to beat. If this child had been placed in a warm bath, and stimulants applied to the epigastrium, and if a little cold water had been dashed in its face, we are of opinion that a sufficient shock would have been applied to rouse it from its insensibility. The inflating the lungs was perfectly right and proper, but other means ought also to have been used in conjunction with it.

The fourth and last chapter is on "Uterine hemorrhage after the birth of the child;" and here we think that the author even outdoes himself. We are, he says, neither to wait until we see "a small stream of blood trickling over the bedside," nor until we are informed "that there is a large discharge." (p. 164.) But we will now give a short view of the author's treatment, and, for obvious reasons, we will do it as much as possible in his own words:

"The first step in the treatment of such cases which the accoucheur should take, is to administer with his own hand, so as to be certain, not less than ʒjss. of laudanum to the patient. The laudanum may be given in a little cold water. In cases of this description we never give less than this quantity, and we have repeatedly given ʒij. as the greatest quantity, and without any disagreeable symptom being produced, farther than occasionally, after the flooding has ceased, the patient has been very sick and vomited, which, however, is rather a favorable result. As soon as the laudanum has been swallowed, a wine-glassful of two ounces of undiluted ardent spirits, whisky, rum, or brandy, should be given to the patient, and the same quantity should be repeated in as rapid succession as the woman can swallow it, until four wine-glassfuls are exhibited. In a few cases we have given the fifth wine-glassful of spirits, but we have never exceeded this quantity, nor do we believe it to be necessary."!! (p. 166.)

Whilst this is doing, the author also directs the external application of cold, according to the usual mode. In his first case the patient took ten ounces of undiluted whisky, in about half the number of minutes; the hand was now introduced, to bring away the placenta, after which the bandage was applied to the abdomen, "when she expressed herself greatly relieved, and *very comfortable.*" (p. 126.) The author remarks, "Although we have not noticed the other usual means, they were all duly put in operation, and should never be neglected." (p. 179.) It is therefore difficult to decide how far the success must be attributed to the cold or to the spirit; we are inclined to think that, beyond making the patient "*very comfortable,*" the spirit had much less to do with making the uterus contract than the cold had.

The author lays great stress on the patient retaining her power of swallowing in these cases, and seems to think it rather extraordinary that this should be the case in the midst of great prostration. "This remarkable state of the organs of deglutition should never be forgotten by the accoucheur, and we cannot too deeply impress it upon his mind; for, as far as we know, it is either not noticed at all, or but imperfectly, by any writer on the subject." (p. 164.) We can only say, in answer to this observation, that if a patient's powers be so far depressed as to render her incapable of swallowing, she can be little else than in articulo mortis. He continues—"We have never seen a case of uterine hemorrhage after the birth of the child in which the woman had not the power of swallowing; but were such a case to occur to us, we should not hesitate to inject a pound of spirits and ʒiij. of laudanum into the bowels, and to retain it by pressure on the fundament."! (p. 189.)

Mr. Craig devotes the last section of his fourth chapter to considering "the opinions and practice of others." With this we shall not take up any more of the reader's time; for, putting aside his peculiar and, we are compelled to add, frequently erroneous views, the whole is written in so disagreeable a spirit, and many of his remarks are so personally rude, that we shall do no more than record our protest against such unwarrantable attacks.

As to his treatment of hemorrhage, after the birth of the child, we need scarcely say that we dissent strongly from it, and cannot help thinking that the number of relapses which he has described would not have taken place if the usual means for stopping hemorrhage had alone been resorted to. He recommends the "frequent introduction of the cold hand," (p. 248,) for the purpose of cooling and emptying the vagina, and considers this "the easiest way to apply cold internally." (p. 249.) Surely the author must be well aware that practitioners have long been in the habit of applying cold to the vagina, for the purpose of stopping a hemorrhage, and by a much simpler means.

---

